# Supporting One Health policies to manage antibiotic resistance in Senegal: a systems analysis using group model building

**DOI:** 10.3389/fpubh.2025.1689609

**Published:** 2025-11-25

**Authors:** Mouhamadou Moustapha Sow, Mamadou Ciss, Nicolas Djighnoum Diouf, Assane Gueye Fall, Ndeye Mery Dia, Tarra Penney, Marion Bordier, Chloe Clifford Astbury

**Affiliations:** 1 Department of Public Health and Social Medicine, Faculty of Health Sciences, Gaston Berger University, Saint Louis, Senegal; 2 Health Science and Research Laboratory, Gaston Berger University, Saint Louis, Senegal; 3 National Laboratory for Livestock and Veterinary Research, Senegalese Institute of Agricultural Research, Dakar, Senegal; 4 Laboratory of Biological, Agronomic, Food Sciences and Modelling of Complex Systems, Gaston Berger University, Saint Louis, Senegal; 5 Global Food System & Policy Research, School of Global Health, York University, Toronto, ON, Canada; 6 Dahdaleh Institute for Global Health Research, York University, Toronto, ON, Canada; 7 Global Strategy Lab, York University, Toronto, ON, Canada; 8 ASTRE, University of Montpellier, CIRAD, INRAE, Montpellier, France; 9 CIRAD, UMR ASTRE, Dakar, Senegal

**Keywords:** antibiotic resistance, One Health, policy, participatory approach, causal loop diagram, Senegal

## Abstract

**Introduction:**

Antibiotic resistance (ABR) is a growing public health issue in Senegal, driven by interconnected factors across human, animal, and ecosystem health. This study applied a participatory systems approach to map the factors influencing ABR in Senegal and identify possible policy actions from a One Health perspective.

**Methods:**

A group model building workshop was held in October 2023 in Dakar with 22 stakeholders from diverse professions and sectors, including human and animal health, environment, agriculture, and food safety. Causal loop diagrams were co-developed to map factors driving ABR and identify intervention points.

**Results:**

The 22 participants identified 55 factors and 88 connections between those factors, that together contribute to the emergence and spread of ABR in Senegal. Four feedback loops were identified: (1) demand for antibiotics; (2) misinformation, public perception and alternative treatments; (3) development of context-appropriate regulations; and (4) enforcement of regulations. Participants proposed 36 actions for ABR mitigation, focusing on: laboratory capacity development; healthcare and infection prevention and control; rational use of antimicrobials in human and animal health; and coordination, communication, and research. Actions considered to have the greatest potential to positively transform the system included: investment in laboratory capacity; enforcement of regulations against the illegal sale of medications; and harmonization of data collection procedures across surveillance systems.

**Discussion:**

This study highlights the value of participatory systems approaches for mapping key drivers of ABR and identifying potential ABR policy actions. While this work integrates cross-sectoral perspectives and provides some actionable insights for evidence-informed decision making, the findings reflect the perspectives of national-level actors and shows strong alignment with international policy and priorities. ABR policy design should also involve local authorities and populations to ensure effective and context-appropriate action. This study provides new empirical evidence to support the development of ABR policy in Sub-Saharan Africa by highlighting the interrelationships between policy areas and the knock-on effects that sectoral and cross-sectoral interventions can have.

## 1 Introduction

Antimicrobial resistance (AMR) has become one of the most pressing global health threats of the 21st century (1). It was responsible for 4.95 million deaths in 2019, with most of the clinical burden borne by low- and middle-income countries (LMICs) (2). AMR is expected to account for 10 million deaths a year by 2050, with long-term economic repercussions (3).

Antibiotic resistance (ABR) is a major contributor to AMR, with ABR alone responsible for 1.29 million deaths worldwide in 2019 (2). Although ABR is a natural phenomenon, misuse of antibiotics in human medicine, animal health and food production is exacerbating its emergence (4, 5). Resistant bacteria and antibiotic residues can spread through the food chain and the environment, making the problem challenging to manage (6, 7). As a result, responsibility for mitigating ABR is distributed across the human, animal and agriculture sectors. ABR is a social-ecological problem whose mitigation requires evidence-informed One Health policies (8). The One Health concept recognizes that the health of humans, domesticated and wild animals, plants, and the wider environment are closely linked and interdependent. It calls for an integrated, unifying approach to sustainably balance and optimize the health of people, animals and ecosystems (9, 10).

ABR poses a significant public health threat in Senegal (11). Bacteria resistant to critical antibiotics have been observed in human and domesticated animal populations, in the food chain, and in wildlife (12–14). Indeed, a 10-year retrospective study in one of the largest healthcare facilities in Dakar reveals worrying rates of Escherichia coli resistance, with 85% resistance to ampicillin and 46% to amoxicillin, both commonly used antibiotics (15). In livestock farming, poultry meat sold in the capital contains bacteria that are resistant to 3rd and 4th generation cephalosporins at rates of 83.9 and 84.4%, respectively (16). In response to this complex problem, Senegal adopted a multisectoral National Action Plan (NAP) to combat AMR in 2017 (17), in line with the World Health Organization's AMR Global Action Plan (18). This NAP covered the period 2018–2022, and a second NAP (developed October 2022–April 2024) was forthcoming at the time of data collection, for the period 2024–2028 (17).

Given the multifaceted nature of ABR, understanding its determinants and identifying effective interventions to mitigate the issue are challenging. A complex systems perspective can provide a holistic view of the system driving ABR and bring to the surface key aspects of systems structure. Through systems mapping processes, participants reflect on and identify key properties of the system they are trying to understand, particularly interrelationships, perspectives and boundaries (19). First, interrelationships indicate how elements in systems are causally interconnected, sometimes by non-linear and time-delayed relationships. These interrelationships can create feedback loops and shape dynamic system behaviors that vary over time, making the behavior of complex systems more challenging to predict. Second, the concept of perspectives underlines how different actors may understand and interpret the system differently, and may also have different priorities in terms of how they would like the system to behave, for example prioritizing health vs. economic outcomes. These different perspectives shape how these actors behave within the system, in turn impacting the system as a whole. Participatory approaches bring actors together to exchange perspectives and create shared understandings of systems structure and behavior. Finally, boundaries in complex systems are artificial and negotiated, used to make a problem manageable to understand and act on. For the system that is driving ABR, different actors may have different perspectives on system boundaries, and the extent to which upstream drivers such as production systems, climate change, conflict or population displacement should be considered a part of the system.

Due to the characteristics outlined above, policies operating within complex systems may be ineffective, provoke unintended consequences or have impacts that vary over time (20). By improving the understanding of systems structure, a systems perspective can therefore support the identification of appropriate and sustainable policy solutions and guide policy decision-making. In short, participatory systems approaches can be useful for integrating stakeholder perspectives on complex problems such as ABR (21–23), improving stakeholders' understanding of system structure, and identifying entry points for effective interventions (24).

Applying this perspective and approach, the aims of this study were to: (i) map the ABR dynamics in Senegal and identify important feedback loops; and (ii) identify priority policy actions to mitigate ABR in the country.

## 2 Materials and methods

### 2.1 Study design and country context

A One Health perspective was used to identify and bring together stakeholders from the different sectors involved in ABR management in Senegal (10). This study used group model building, a workshop-based approach to systems modeling (25, 26). This method was chosen as being appropriate for our research aims, as it focuses on developing a shared understanding of a multi-sectoral system in the form of a map, allowing stakeholders to clearly situate themselves within the system and consider how to collaborate more effectively to address shared problems. During the workshop, stakeholders co-developed causal loop diagrams (CLDs), a form of qualitative systems map, and identified priority actions for ABR mitigation. The workshop was organized in October 2023, at which time the term of Senegal's first AMR NAP had just come to an end and there was a need to produce evidence to inform the development of the second NAP covering the period 2024–2028 (17).

The study was designed and carried out by a team composed of Canadian, French and Senegalese researchers, working in a variety of disciplines (health policy, epidemiology, mathematics, public health, and microbiology) and sectors (human health, animal health, and food systems). The team's disciplinary expertise is complemented by a thorough command of participatory methods, systems thinking and ABR.

### 2.2 Participant recruitment

We recruited targeted stakeholders from central-level institutions involved in ABR governance and related policies. We followed a two-stage process to identify potential participants. First, we collected the names of the 67 institutions constituting the AMR thematic group of the One Health national platform, which is the intersectoral coordinating mechanism for One Health governance in Senegal (27). This list was reviewed by three ABR policy experts in order to select the most relevant institutions for ABR management to invite to the workshop. We applied the following inclusion criteria for participant recruitment: participants' institution must be (i) a member of the One Health platform's Thematic Working Group (TWG) on AMR, (ii) actively involved in the development of One Health policies and interventions, and (iii) implementing or supporting priority interventions of first AMR NAP. Participants were excluded if their institution was (i) absent from the TWG, and/or (ii) not involved in the process of formulating and implementing priority policies and interventions for the AMR NAP. At the end of this process, we obtained a list of 29 institutions (four operating at regional level and 25 at national level) covering the sectors of human health, animal health, environmental health, and agriculture and food safety. An invitation to participate in the workshop was sent to each of the institutions on 9 October 2023.

### 2.3 Data collection

Data collection took place during a 2-day workshop held on 25–26 October 2023 in Dakar. Workshop activities were defined based on published guidance around best practices for developing and facilitating group model building sessions, using standardized scripts to run each part of the workshop (25, 26, 52, 54). Given their high number, participants were split into three groups to carry out the activities, ensuring that all sectors were represented in each group. Each group's discussion was supported by a facilitator and a note-taker from the research team. The facilitation team was composed of senior and junior researchers with expertise in the Senegalese context. Prior to the workshop, the facilitation team participated in a 2-day in-person training, focusing on introducing key concepts relating to complex adaptive systems; the group model building methodology; and facilitation steps and strategies. This involved a mock workshop in which facilitators participated in group model building activities themselves, and review and adaptation of workshop materials (e.g., introductory presentation). This training added to facilitators' existing content expertise in One Health and ABR. A team member with expertise in systems approaches (CCA) led the training and was also on-site during the workshop to support the facilitation as needed.

In each group, participants followed six steps, described in Table 1. This structured approach encourages discussion among participants at each stage and builds consensus for each element in the system, thereby enhancing inclusive participation and data quality through an iterative process (28). After the introductory presentation, participants used the graphs-over-time exercise to illustrate their perception of how ABR in Senegal was evolving. Then, participants used sticky notes to propose and discuss variables, i.e., factors contributing to the emergence and spread of ABR in Senegal. Next, the causal connections between these factors were identified and represented on the map using arrows, following standard notation (Table 2). These represent causal connections theorized by the participants, reflecting their understanding of the system in which they were engaged based on their professional experience and knowledge. If an increase in one factor caused an increase in a second factor (or a decrease in one caused a decrease in the other), a positive (+) sign was associated with the arrow. Conversely, if an increase in one factor caused a decrease in a second factor (or a decrease led to an increase), a negative (–) sign was added to the arrow connecting the two, and the shaft drawn as a dotted line. Participants did this in two phases, first developing a connection circle to generate ideas about elements and connections, then building on this work to identify system structures including feedback loops and long causal pathways. Given participants' differing professional backgrounds and experiences, discrepancies sometimes arose regarding the variables and connections that they identified. In each stage, participants had the opportunity to individually reflect on the system elements they considered important, then propose them to their groups. Where other participants did not agree with the variable or connection being included in the system, participants could discuss the element in question to try to seek consensus. During these discussions, facilitators asked probing questions to help participants clarify their perspectives and arguments. Elements were only included on the map when there was a consensus agreement to include them. In line with methodological guidelines, facilitators proposed that elements be “parked” and returned to later in the discussion when elements were discussed at length with no sign of consensus being reached.

**Table 1:** Description of scripts used to guide group model building activities.

**Script**	**Description**
Introductory presentation	Research team briefly presents systems thinking and group model building, to help participants understand the necessary terminology, notation and process.
Graphs over time	Participants consider the dynamic behavior of ABR in Senegal over time, and agree on a *status quo* prediction for how ABR is likely to evolve (known as the reference mode)
Variable elicitation	Participants identify variables that influence ABR, beginning with individual brainstorming on sticky notes. Participants then take turns to propose variables to the group. Variables are selected if they are consensually considered relevant by the group.
Connection circle	Participants create a circle using the variables they have identified and propose causal connections between them as an intermediate step to creating a causal loop diagram, allowing participants to start generating ideas about elements and connections.
Causal mapping	Participants integrate the variables and connections identified previously to develop a CLD, referring to the reference mode to determine whether the CLD explains the reference mode, and iterating their model as needed.
Action ideas	Participants identify priority actions for ABR mitigation, brainstorming actions individually, then having a consensus-based discussion on how feasible actions are, and whether actions are likely to have a shallow or deep impact.

**Table 2:** Overview of CLD notation.

**Name**	**Visualization**	**Description**
Relationship with positive polarity	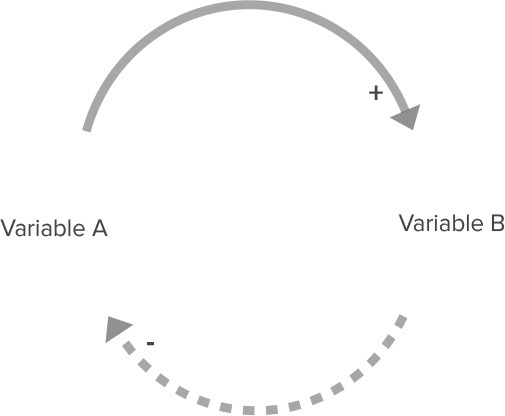	An increase in variable A causes an increase in Variable B. A decrease in Variable A causes a decrease in Variable B.
Relationship with negative polarity	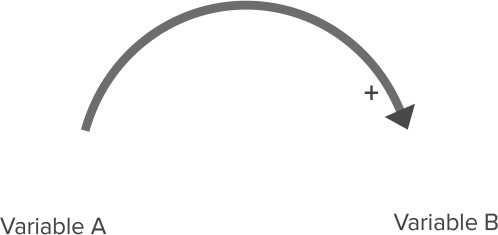	An increase in variable A causes a decrease in Variable B. A decrease in Variable A causes an increase in Variable B.
Reinforcing feedback loop	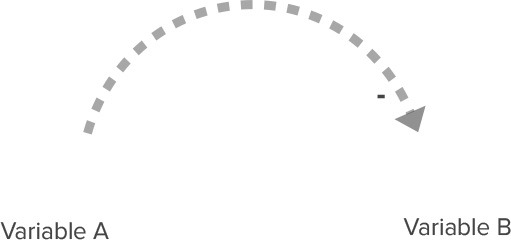	Two relationships with the same polarity (in this case, two relationships with a positive polarity) create a closed circle of cause and effect. This can lead to either increasing growth or increasing decline in these variables.
Balancing feedback loop	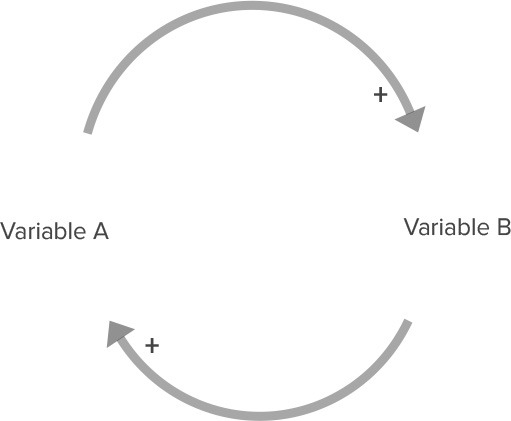	Two relationships with different polarities (i.e., one relationship with positive polarity and one relationship with negative polarity) create a closed circle of cause and effect. This leads to a balancing effect, where the feedback between variables leads to stabilizing behavior.

Finally, based on the representation of the ABR problem they had constructed and their knowledge of the context, participants identified priority actions to manage ABR, and classified them as either “shallow” (action easy to implement but with little potential to transform the system) or “deep” (action difficult to implement but having a great potential for transformation). This classification was completed by participants themselves, based on their professional experience and their perspective on which actions would be positioned to have more or less transformative impacts on the system they had mapped as a group. Participants completed this exercise as part of the “action ideas” activity (see Table 1), which is an activity commonly used in group model building workshops, informed by Meadows' classification of leverage points for actions in complex systems (24).

At the end of each activity, plenary discussions allowed groups to share their work and provided opportunities for discussion, clarification and refining ideas. At the end of the workshop, pen-and-paper CLDs developed by each of the three groups were collected by the research team for analysis. The research team also collected other workshop outputs for analysis, namely the proposed actions classified by feasibility and impact, and handwritten notes taken by the note-takers during the workshop.

### 2.4 Data analysis

First, workshop outputs (i.e., the CLDs developed by the three groups and additional information collected by the note-takers) were entered into the French version of the online mapping platform Kumu (29) by the research team shortly after the workshop. After harmonizing variable names and removing duplicate variables, all variables and connections from the three groups were combined into a single CLD, with factors visually represented by sector (i.e., human health, animal health, environment, agriculture, socio-economic, and cross-sectoral) using color codes. Where connections were in conflict (i.e., groups considered that two variables were connected, but disagreed on the type of connection), connections would be noted as “equivocal,” as has been done in previous published examples (30), though in practice no conflicts of this type were identified between groups. The feedback loops were identified by the research team based on information from the workshop and an overall analysis of the final CLD. Kumu was used to create a visualization of each of the feedback loops and their proximal factors (i.e., system elements driving or impacted by the feedback loop).

In a second step, the research team also examined the actions proposed by each group and integrated them into a single list, reflecting their classification by participants as shallow or deep. The actions were then classified according to the four policy areas of action of the first national AMR action plan (2018–2022), which also serve as the basis for the development of the second plan (2024–2028): (i) laboratory capacity development; (ii) healthcare, and infection prevention and control; (iii) rational use of antimicrobials in human and animal health; and (iv) coordination, communication, and research (17).

### 2.5 Ethics approval and consent to participate

This study was approved and authorized by the Senegalese National Ethics Committee for Health Research (*Comité National d'Ethique pour la Recherche en Santé*, or CNERS) in January 2023 (N°019MSAM/CNERS/SP).

## 3 Results

### 3.1 Participant characteristics

The workshop was attended by 22 representatives of 21 institutions, representing a 76% response rate to invitations (see Supplementary File 1). Four regional and four national institutions declined the invitation due to scheduling conflicts. The two most-represented sectors were human and animal health (each with *n* = 6), and agriculture and food safety (*n* = 6), followed by environmental health, (*n* = 2) and multisectoral (i.e., coordinating staff from the national One Health platform; *n* = 2). The participants were from governmental authorities (*n* = 10), technical and research institutes (*n* = 5), intergovernmental organizations (*n* = 3), and the private sector (*n* = 4). Participants' disciplinary perspectives included epidemiology, microbiology, legal sciences, veterinary and human medicine, environmental sciences, agricultural sciences, communication, public health, political science and sociology. There was a gender balance among participants (11 women and 11 men).

### 3.2 Dynamic behavior of ABR in Senegal over time

In the workshop, there was a consensus among participants that ABR in Senegal was on the rise, and that this was likely to continue given current conditions. However, there were debates about the extent and speed of ABR emergence and spread.

### 3.3 Systems map of antibiotic resistance and identified feedback loops

**Figure 1:**
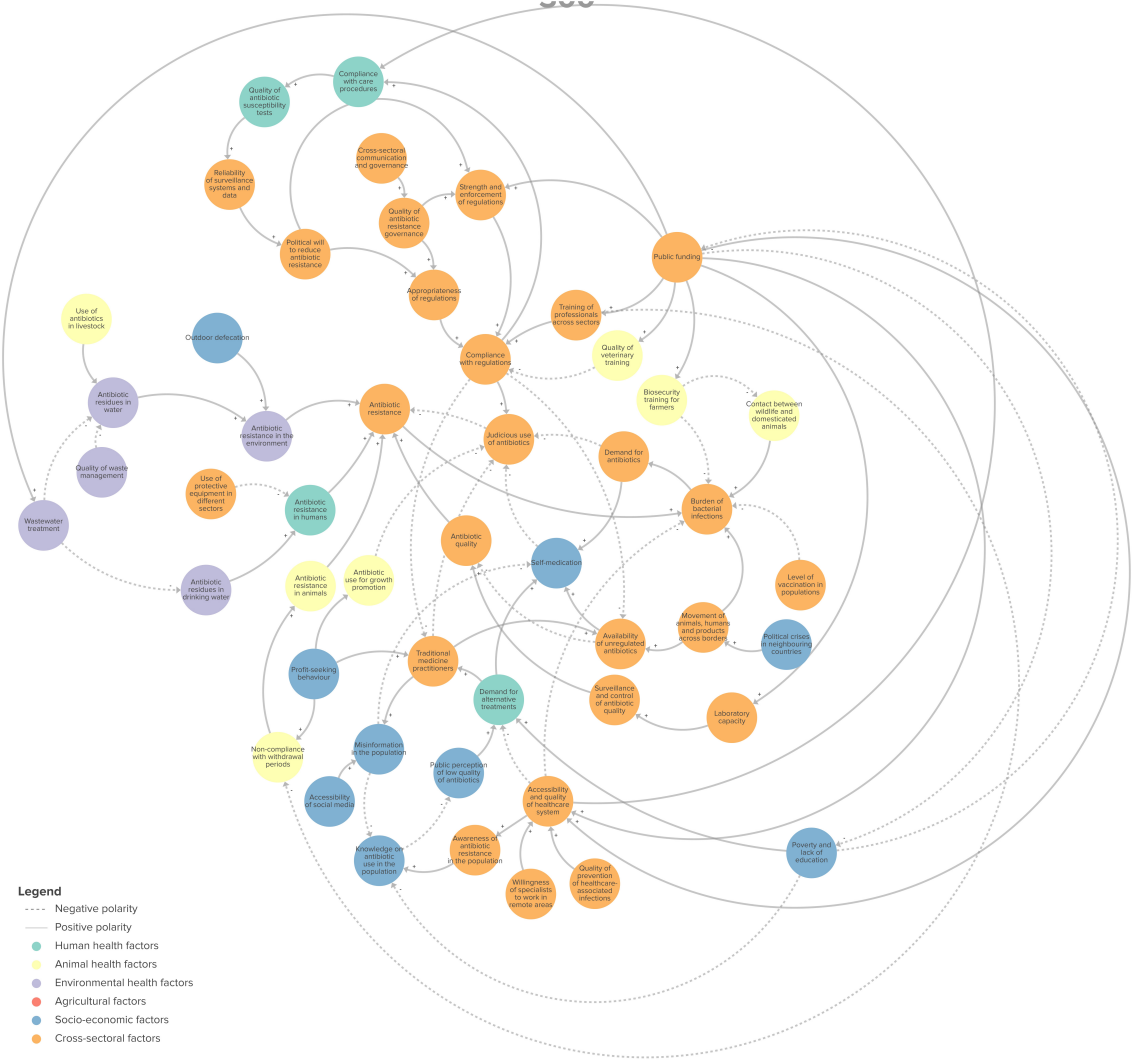
Overview of the structure of the CLD representing the emergence and spread of antibiotic resistance in Senegal.

The CLD co-developed by participants consists of 55 variables and 88 connections. Figure 1 provides an overview of the structure of the CLD. It can be explored in detail online: https://kumu.io/chloe-ca/design-amr-senegal.

These variables were classified as animal health (*n* = 7), environmental health (*n* = 5), human health (*n* = 4), agriculture (*n* = 5), socio-economic (*n* = 9), and cross-sectoral (*n* = 25).

**Figure 2:**
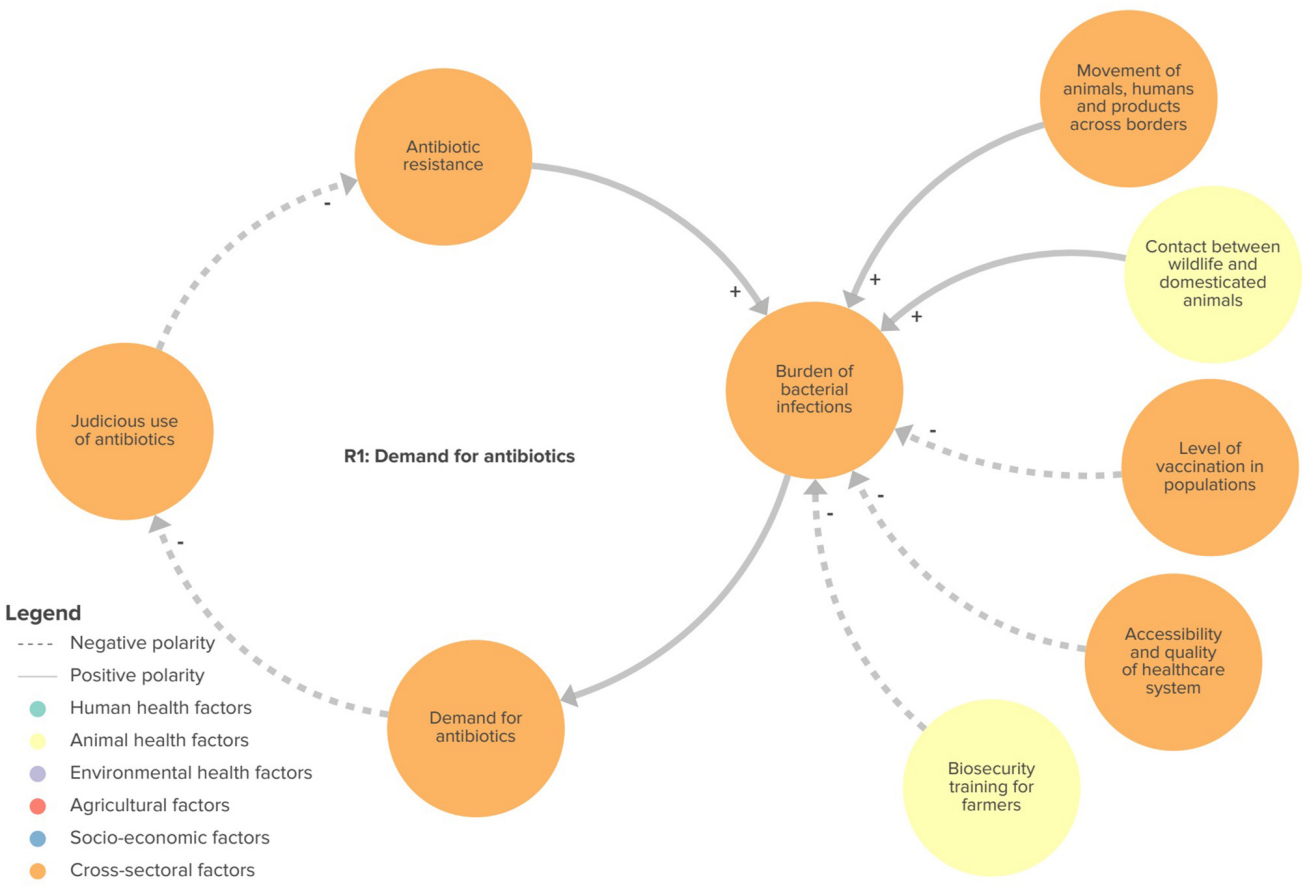
Feedback loop related to the increasing demand for antibiotics. R1, reinforcing loop 1.

**Figure 3:**
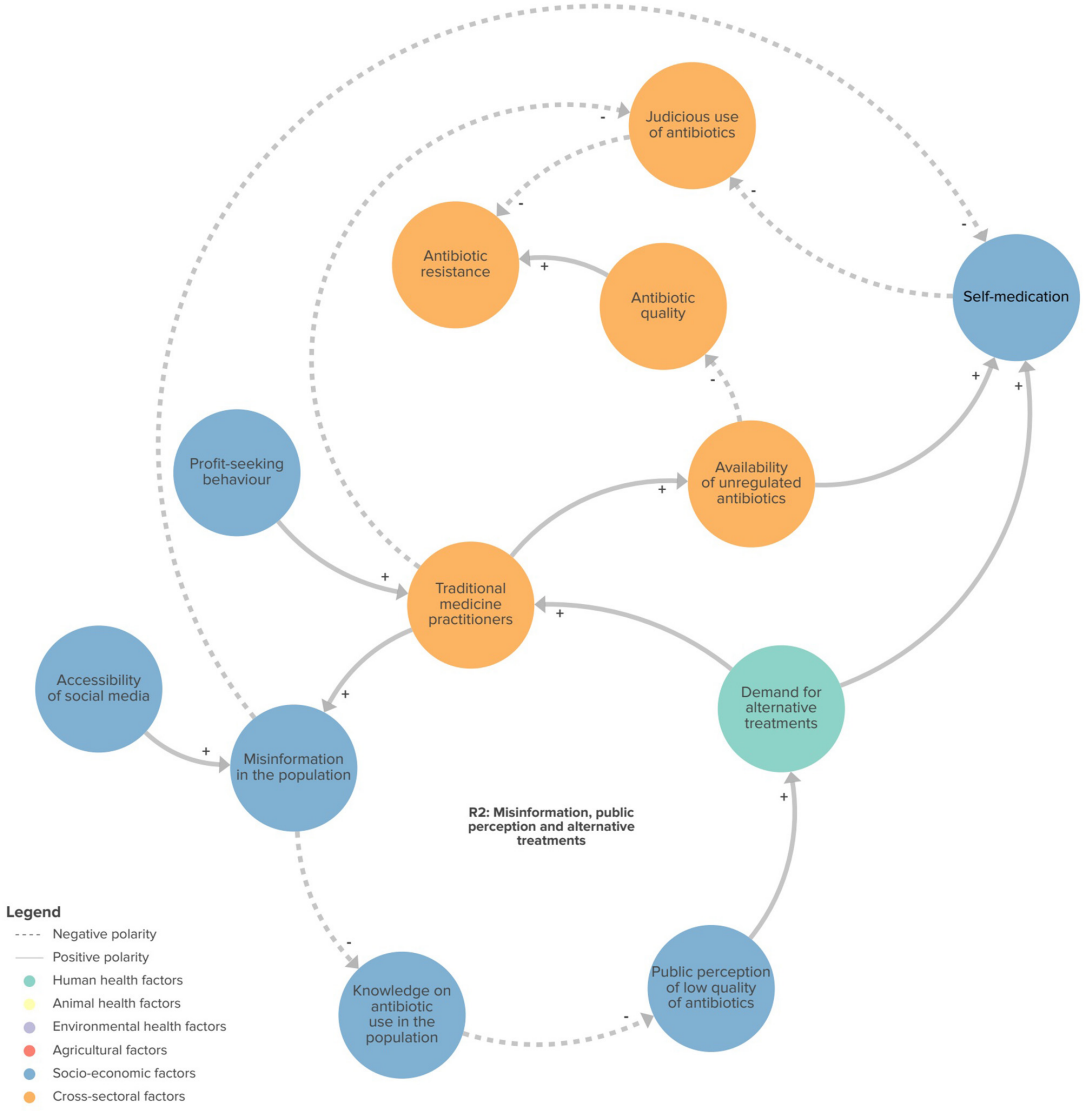
Feedback loop related to misinformation, public perception and alternative treatments. R2, reinforcing loop 2.

**Figure 4:**
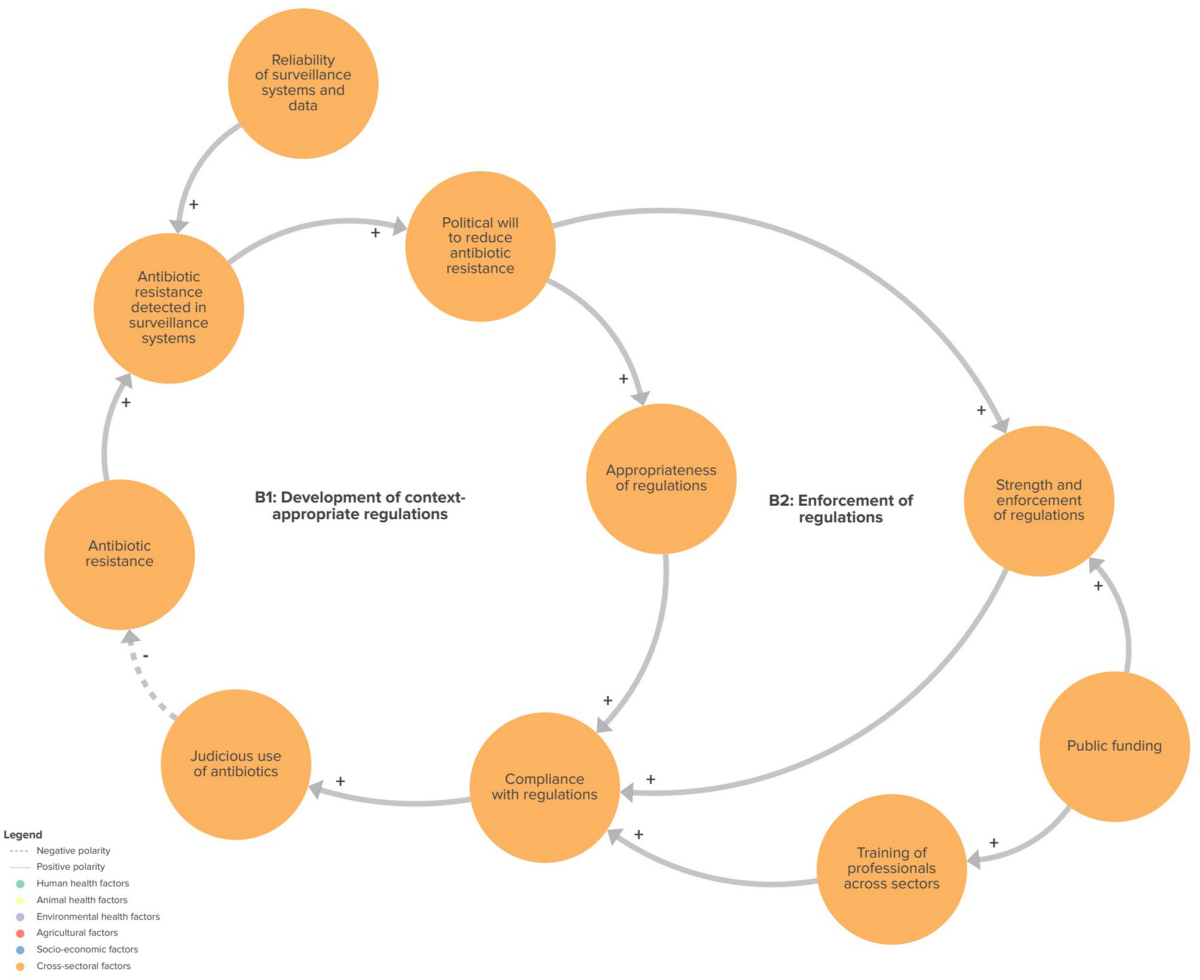
Feedback loops related to developing and enforcing context-appropriate regulations. B1, balancing loop 1; B2, balancing loop 2.

We identified four feedback loops in the CLD that may play a role in ABR: (1) demand for antibiotics (R1 in Figure 2); (2) misinformation, public perception and alternative treatments (R2 in Figure 3); (3) development of context-appropriate regulations (B1 in Figure 4); and (4) enforcement of regulations (B2 in Figure 4). Of the four loops we identified, the first two were reinforcing feedback loops driving an increasing burden of ABR, highlighting important areas for action. The two remaining feedback loops were balancing feedback loops, illustrating the potential of regulation and surveillance, in relation to the political will to mitigate ABR.

#### 3.3.1 Demand for antibiotics

Participants considered the increase in ABR in Senegal to be closely linked to the high demand for antibiotics (Figure 2). This demand was perceived as mainly driven by the high prevalence of bacterial infections in human and animal populations.

Two key drivers of bacterial infections were identified: contact between wildlife and domesticated animals, and the movement of animals, humans and agricultural and livestock products across borders. Participants underlined the significant contact between domesticated and wild animals, especially between migratory birds and local poultry. This close contact, combined with poor biosecurity practices and low vaccination coverage, are increasing the risk of the emergence and spread of animal diseases, which in the case of zoonotic diseases can also be transmitted to humans. This increases the use of antibiotics, often injudicious, especially in areas lacking human and animal health facilities. In addition, participants noted that the intense movements of animals and people from neighboring countries, especially during religious holidays (Tabaski, Gamou, Christmas and Easter) facilitate the introduction of bacterial diseases into the country. These movements are eased by Senegal's membership of two regional economic communities [the Economic Community of West African States (ECOWAS) and the West African Economic and Monetary Union (WAEMU)], within which movement of goods and persons are facilitated, and by the poor enforcement of regulatory border controls.

#### 3.3.2 Misinformation, public perception and alternative treatments

Antibiotic misuse in human and veterinary medicine was reported to be driven by a range of primary variables, including the population's poor knowledge about antibiotic usage, access to an informal pharmaceutical market, the existence of unregulated and influential health practitioners, and misinformation about antibiotic use from social media (Figure 3).

Participants linked the misuse of antibiotics in Senegal to the demand for traditional medicine practitioners who provide an alternative to regulated medical care. They perceived the population as having a low level of knowledge about the use of antibiotics, especially with regard to the importance of following prescriptions. As a result, treatments could be ineffective, leading to a perception of antibiotic ineffectiveness within certain communities. This could drive these communities to seek alternative treatments from traditional practitioners, who in many cases offer lower-quality antibiotics with false labeling, and prioritize profit at the expense of public health. Participants also noted the role of social media in promoting misinformation about the use of antibiotics, encouraging people to turn to unregulated treatments through uncontrolled advertising. Literacy rates are relatively low in Senegal, so audio or visual information is popular in many communities. As such, short videos on TikTok and WhatsApp audio messages, which can be easily shared between users, are frequently relied on for information, and can propagate misinformation on the use of antibiotics. Participants provided examples in livestock farming, where social media allows the formation of communities of farmers who provide advice to each other regarding disease management, without the supervision from an animal health professional, contributing to the misuse of antibiotics.

#### 3.3.3 Development and enforcement of context-appropriate regulations

Context-appropriate and well-enforced regulation, supported by strong political will, was seen as key to ensure appropriate access to and use of antibiotics, and to mitigate ABR risk (Figure 4).

However, according to participants, the lack of human and financial resources, combined with high levels of corruption, compromises the rigorous enforcement of regulations on the importation of pharmaceuticals, possession, sale and use. This contributes to the existence of the informal market and the proliferation of inappropriate practices with regard to antibiotic use. Public funding was seen as an essential factor to strengthen regulatory enforcement and enable the training of professionals in pharmaceutical regulation.

Participants saw surveillance as important in providing tangible evidence of the prevalence of resistance and its impacts, including on public health and the national economy, to appropriately inform policy development. The provision of high-quality surveillance data was considered to be a mechanism to generate more political will to combat ABR. This increase in political will would reinforce sectoral surveillance and support integrated surveillance, which would in turn generate more comprehensive and accurate data to better inform policy development.

While this highlights a potential mechanism for ABR mitigation, participants' perception that ABR is still on the rise in Senegal suggests that this balancing loop is not yet powerful enough to counterbalance other dynamics in the system. Data on antibiotic use and resistance are not always available due to weaknesses in human, animal and agri-food surveillance systems. The institutions and laboratories responsible for this task face staff shortages and supply disruptions that prevent them from systematically collecting and analyzing data.

### 3.4 Identification of priority actions

During the workshop, participants identified 36 priority actions that could mitigate ABR in Senegal, classifying 22 as shallow and 14 as deep across the four domains considered: (1) laboratory capacity development (*n* = 6); (2) healthcare, infection prevention and control (*n* = 9); (3) rational use of antimicrobials in human and animal health (*n* = 5); and (4) coordination, communication, research (*n* = 16).

For each domain both shallow and deep actions were identified, except in the domain of rational use of antimicrobials in human and animal health, where only deep actions were proposed. Table 3 presents illustrative examples of the deep and shallow actions identified by participants across policy domains. The full list of proposed actions is available in Supplementary File 2.

**Table 3:** Examples of actions in the different domains, with their classification in terms of shallow or deep leverage point.

**Policy domain of the NAP**	**Example of actions**	**Action category**
Laboratory capacity development	•Strengthening laboratory equipment •Developing harmonized data collection support for antibiotic resistance	Shallow deep
Healthcare, and infection prevention and control	•Strengthening health human resources in remote areas •Ensuring equitable access to medical care	Shallow deep
Rational use of antibiotics in human and animal health	•Mapping, harmonizing and updating regulations on antibiotics use •Enforcing sanctions against the illegal sale of antibiotics and strengthening criminal penalties	Shallow deep
Coordination, communication, research	•Mobilizing resources for action on antibiotic resistance •Harmonizing antibiotic resistance actions across sectors	Shallow deep

The key actions proposed by participants for each of the four domains are summarized below.

#### 3.4.1 Laboratory capacity development

Developing laboratory capacity is central to strengthening surveillance, which was considered key to develop appropriate policy solutions and foster political will for ABR management. Harmonization of data generated by laboratories across surveillance components could allow their centralization and joint analysis to provide relevant information for formulating evidence-informed policies, strategies and interventions. Improving surveillance requires provision of laboratories with reagents for systematic bacterial isolation and antibiotic susceptibility testing, when a bacterial infection is suspected and before prescribing antibiotics. Participants also expressed the need to extend surveillance to other domains, such as antibiotic residues in food of animal origin.

#### 3.4.2 Healthcare, and infection prevention and control

Participants identified two areas for improving access to quality health services: strengthening health services in terms of equipment and materials, and appointing well-trained healthcare staff in remote areas. According to workshop participants, this would support equitable access to healthcare for the whole population and reduce antibiotic misuse and informal traditional practices. In addition, participants underscored the importance of revitalizing hospital-acquired infection control committees as a means of reducing exposure to ABR. These committees are responsible for developing and implementing good hygiene practices in healthcare facilities and for the collective management of biomedical waste, with the aim of reducing infection, including by resistant bacteria.

#### 3.4.3 Rational use of antibiotics in human and animal health

Across the system, participants identified the need to strengthen regulation to ensure the rational use of antibiotics in the human and animal health sectors. This includes enforcing compliance with antibiotic withdrawal periods in animal production to reduce antibiotic residues in food products, and regulating and supervising antibiotic use, particularly among community health workers for humans and animals who are the first providers of health services in rural areas. Furthermore, strict control of the informal market for pharmaceuticals, including antibiotics, was considered essential to eradicate unregulated medical practices. To achieve this, participants proposed mapping existing texts, identifying regulatory gaps and updating regulation to ensure it is appropriate for ABR mitigation.

#### 3.4.4 Coordination, communication, research

Participants recommended the strengthening of One Health governance mechanisms by focusing on coordination, communication and pooling efforts across sectors. This would make actions more effective and less costly. Effective One Health governance mechanisms may contribute to improving communication between stakeholders and increasing the use of information on ABR. They also proposed training, awareness-raising and communication campaigns aimed at the general public and professionals in rural and urban areas to improve the rational use of antibiotics, emphasizing that these programmes should be adapted to their respective audiences. However, participants disagreed regarding the impact of these actions, given past experience: Some argued that educational interventions could be truly transformative, while others stated that the dissemination of information was often insufficient to change behavior at a population level, particularly in the absence of accessible basic health structures and quality antibiotics in remote areas.

## 4 Discussion

The aim of this study was to map the system that contributes to ABR emergence and spread in Senegal, and to identify policy actions to mitigate ABR at country level. Participants identified ABR as being driven by factors that span the human, animal, and environment sectors and that interact with socio-economic factors, highlighting the value of the One Health perspective in addressing ABR. The CLD developed by participants included four feedback loops: (1) demand for antibiotics (2) misinformation, public perception and alternative treatments; (3) development of context-appropriate regulations; and (4) enforcement of regulations. Reflection on these dynamics prompted participants to identify potential policy actions for mitigating ABR, which we classified into four policy areas: (i) laboratory capacity development; (ii) healthcare, and infection prevention and control; (iii) rational use of antimicrobials in human and animal health; and (iv) coordination, communication, research.

Some of the drivers identified in this analysis have been found in other studies in the Sub-Saharan African context. In this region, it has been evidenced that ABR is favored by poverty, lack of education, staff shortages in healthcare systems, inadequate national and regional surveillance networks, and inappropriate regulation for antibiotic access and quality (31). The high bacterial pressure, through the endemicity of a large number of diseases such as bacterial respiratory infections, meningitis and diarrhea, has also been described in the literature as a major factor contributing to high demand for antibiotics in West Africa, both for prevention and treatment (32). During our workshop, participants identified strong links between the circulation of false information about antibiotics on social media and the persistence of their inappropriate use. As a result, antibiotics of lower quality are advertised and sold without a prescription, even though drug regulations in Senegal and other WAEMU country members only allow certified professionals to prescribe pharmaceuticals (33). The democratization of information through social media has also been recognized as accelerating the spread of misinformation and rumors about antibiotics and the proliferation of unregulated online pharmacies in other African nations (34).

In this study, our main focus was not the potential for transmission of ABR and antibiotic residues between human and animal populations through livestock farming, aquaculture, and slaughterhouses, as this has been underlined in previous studies conducted in Burkina Faso and Senegal (35, 36). Given the nature of the participants who attended the workshop, the focus was more strongly on overarching policy issues such as regulation, surveillance and governance mechanisms. We also identified feedback loops that explain ABR dynamics in Senegal. These highlight potential areas for actions to reduce the emergence and spread of ABR. Through the workshop process, stakeholders proposed actions that could potentially bring about systemic changes, some superficial and some profound.

However, workshop outputs must be understood in context. The results represent the views of central-level stakeholders, who in many cases have a long history of working with, and sometimes for, international organizations and health programmes. As such, their perspectives may be broadly aligned with dominant international discourse framing antimicrobial resistance, including ABR, as a high-priority threat to human health security and economic progress, with low- and middle-income countries as hotspots (37, 38). This bias has been identified by Ridde in Burkina Faso, whereby the discourse and arguments of health system actors are constructed to align with the perspectives and expectations of international organizations rather than to reflect local realities (39). The major challenges facing local populations are related to access to basic primary care and standard medicines. The health system infrastructure remains highly fragmented, focused on hospitals and curative care, which are generally found in urban areas (40). Furthermore, these inequalities are perpetuated by the unwillingness of a large proportion of healthcare professionals to practice outside the capital, Dakar, which alone accounts for 43% of human resources in the healthcare sector, highlighting geographical inequities (41, 42). In addition, funding for the health sector has long remained at around 5% of the national budget (43). In some areas of the country, the informal market is unofficially at the heart of healthcare systems. People living in precarious situations resort to medicines, especially substandard antibiotics sold on the streets, contributing to the risk of inappropriate use of antibiotics and thus constituting a public health problem (44).

In their conscious or unconscious effort to align with the international vision, workshop participants may have proposed actions that do not necessarily consider the uniqueness and history of the areas where they will be implemented, nor the complexities of issues dealt with at the local level (45). This is what Dibakana calls the “paradox of the internationalization of health policies in Africa,” whereby health policies supported by international organizations are not adequately adapted to local contexts (46).

This study produced information to guide policy design in the context of the development of Senegal's second AMR NAP. The results produced during the workshop will likely inform the contents of this second NAP: since the same actors will be involved in its drafting, it will be relatively easy for them to take up and defend positions they have negotiated during a previous participatory process (47). However, as described before, the workshop participants do not carry all of the same perspectives and realities as local actors in the field. In addition, the policy design workshops for the second NAP will be organized with the support of international partners and guided by their desire to comply with international standards, such as the International Health Regulations. Consequently, the NAP may not be adequately adapted to local contexts, and the defined actions, even if implemented, may not trigger a drastic reduction in ABR in the country.

This has been observed in many sub-Saharan countries that have developed a NAPs to combat AMR with strong influence from international partners and that have faced multiple financial and institutional obstacles during their implementation because of a lack of ownership by national actors or insufficient dedicated domestic resources (39, 40, 53). In addition, no single action constitutes a miracle solution for combating ABR. The implementation of systemic interventions must take into consideration socio-economic dynamics, and avoid focusing solely on downstream factors without taking sufficient account of local contexts such as poverty and the lack of basic health infrastructure (48, 49), or the rapidly changing context in which the problem occurs (50). Furthermore, One Health policies in low- and middle-income countries would benefit from greater involvement of and consultation with populations, decolonizing health through an overhaul of how programmes are governed, funded, formulated, implemented and evaluated, and addressing socio-economic health inequities and power dynamics (51). This underlines the importance of continuing this participatory systems modeling work with local actors and contextual experts to gather their perspectives and to integrate them when translating national policy objectives into local actions. As well as engaging local actors, future work to guide evidence-informed ABR policy in Senegal could focus on evaluating these policies as they are implemented, to determine how they are impacting key indicators across One Health sectors.

## 5 Conclusion

Using a participatory systems approach guided by a One Health perspective, stakeholders from multiple sectors produced a map of the factors contributing to the emergence and spread of ABR in Senegal. Within this map, we identified four feedback loops driving the evolution of ABR: (1) demand for antibiotics (2) misinformation, public perception and alternative treatments; (3) development of context-appropriate regulations; and (4) enforcement of regulations. Reflecting on the structure of the system, participants proposed priority actions for ABR mitigation, including actions they perceived as having the capacity for deep transformation of the system based on their experience working within this shared system. These included strengthening One Health governance mechanisms, enforcing regulations, making healthcare systems accessible country-wide, and raising awareness to change behavior. Integrating this systems understanding and stakeholder proposals for transformative actions into Senegal's second NAP would support more inclusive, sustainable and effective ABR policy and governance.

However, the effective translation of these policy objectives into concrete and appropriate actions depends on integrating the needs and realities of local authorities and populations. A participatory approach to develop policy solutions at the local level would help reduce the adverse effects of top-down policies while promoting context-appropriateness. This engagement of local actors is an essential step, particularly in low- and middle-income countries such as Senegal that may have less power in global agenda-setting and policymaking.

## Data Availability

The original contributions presented in the study are included in the article/Supplementary material, further inquiries can be directed to the corresponding author.
