# Supplementary material for: Supporting One Health policies to manage antibiotic resistance in Senegal: a systems analysis using group model building
Source: Front Public Health. 2025 Nov 25;13:1689609. doi: 10.3389/fpubh.2025.1689609 (PMC12687748; doi:10.3389/fpubh.2025.1689609)
Supplement: Supplementary file 1 [file Table_1.docx]

Supplementary file 1

List of institutions invited and participating to the workshop

| Level | Structures | Sector | Typology | Participation |
| --- | --- | --- | --- | --- |
| National | High national council for global health security | Cross-sectoral | Governmental  authorities | Present |
| National | Veterinary Services Department | Animal health | Governmental  authorities | Present |
| National | National Codex Alimentarius Committee | Agriculture and  food safety | Technical and  research institutes | Present |
| National | National Supply Pharmacy | Human health | Governmental  authorities | Present |
| National | Livestock Department | Animal health | Governmental  authorities | Present |
| National | Plant Protection Department | Agriculture and  food safety | Governmental  authorities | Present |
| National | National Medical Council | Human health | Professional  organizations | Present |
| National | National Pharmacists Council | Human health | Professional  organizations | Absent |
| National | National Dental Surgeons Council | Human health | Professional  organizations | Absent |
| National | National Veterinarians Council | Animal health | Professional  organizations | Present |
| National | Environment and Classified Establishments Department | Environmental  health | Governmental  authorities | Present |
| National | Fisheries Industries and Processing Department | Agriculture and  food safety | Governmental  authorities | Present |
| National | Institute of Food Technology | Agriculture and  food safety | Technical and  research institutes | Present |
| National | National Public Health  Laboratory | Human health | Technical and  research institutes | Present |
| National | National Council of the Breeders' Association | Animal health | Professional  organizations | Present |
| National | CEVA Animal Health | Animal health | Private sector | Present |
| National | Senegalese Institute for  Agricultural Research | Agriculture and  food safety | Technical and  research institutes | Present |
| National | Senegalese Pharmaceutical  Regulatory Agency | Human health | Governmental  authorities | Present |
| National | Association Private laboratory | Human health | Private sector | Absent |
| National | National Parks Directorate | Environmental  health | Governmental  authorities | Absent |
| National | Sanitation Department | Environmental  health | Governmental  authorities | Present |
| National | National Food Analysis and Control Laboratory | Agriculture and  food safety | Technical and  research institutes | Absent |
| Regional | West African Health Organization (WAHO) | Human health | Regional partner | Absent |
| Regional | Regional Centre for Animal Health | Animal health | Regional partner | Absent |
| Regional | West African Economic and Monetary Union | Animal health | Regional partner | Absent |
| International | USAID | Animal health | International  partner | Present |
| International | Food and Agriculture  Organization | Agriculture and  food safety | International  partner | Present |
| International | World Health Organization | Human health | International  partner | Present |
| International | International Livestock  Research Institute | Animal health | Technical and  research institutes | Present |
