# Supplementary material for: Supporting One Health policies to manage antibiotic resistance in Senegal: a systems analysis using group model building
Source: Front Public Health. 2025 Nov 25;13:1689609. doi: 10.3389/fpubh.2025.1689609 (PMC12687748; doi:10.3389/fpubh.2025.1689609)
Supplement: Supplementary file 2 [file Table_2.docx]

Supplementary file 2

List of interventions proposed by the participants

| Low impact /  Easy to achieve | Low impact /  Difficult to achieve | High impact /  Easy to achieve | High impact /  Difficult to achieve |
| --- | --- | --- | --- |
| - Promoting sanitation in local areas - Raising awareness among stakeholders - Monitoring and quality control of medicines - Communicating the risk of antibiotic resistance to the population | - Combating fraudulent and counterfeit medicines | - Set up nosocomial infection control committees and make them operational - Include antibiotic resistance training in student curricula - Popularize antibiotic resistance for the general public and professionals - Strengthen laboratory, control and analysis equipment - Promote education for stakeholders at grassroots level - Create a national network of professionals - Publicize the laws and regulations in force - Set up a system to monitor the presence of antibiotic residues in foodstuffs - Give greater responsibility to producers' organizations in the fight against antibiotic resistance - Provide health services with equipment and materials throughout the country - Provide reagents to bacteriology laboratories (antibiotic discs, culture media) - Capitalize on information on antibiotic resistance at the level of service delivery points - Pool all actions relating to antibiotic resistance by all stakeholders | - Ensuring equitable access to care - Promote data research on antibiotic resistance - Implementing an integrated biomedical waste management system - Mobilize resources - Equip public health and research facilities - Map, harmonize and revitalize regulatory texts - Communicate about antibiotic resistance to the public - Develop harmonised data collection support for antibiotic resistance - Draw up a communication plan on antibiotic resistance - Have kits for antibiotic susceptibility testing - Strengthen strategies for controlling the sale of medicines - Build the capacity of players in the field of antibiotic resistance and the One Health approach - Make healthcare services accessible - Enforce regulations on waiting times - Raise public awareness of antibiotic resistance - Enforce sanctions against the illegal sale of medicines and strengthen criminal sanctions - Strengthen human resources in remote areas |
